# Phosphatase and tensin homolog: A potential target for therapeutic intervention in optic nerve regeneration

**DOI:** 10.4103/NRR.NRR-D-24-01599

**Published:** 2025-09-19

**Authors:** Bin Tong, Yanzhuo Song, Zhengyang Li, Muhan Cai, Haodong Qi, Kangtai Su, Hong A. Xu

**Affiliations:** 1 School of Ophthalmology and Optometry, The Huankui Academy, The First Clinical Medical College, School of Basic Medical Sciences, The Second Affiliated Hospital, and Institute of Biomedical Innovation, Jiangxi Medical College, Nanchang University, Nanchang, Jiangxi Province, China; 2 Jiangxi Province Key Laboratory of Brain Science and Brain Health, Nanchang, Jiangxi Province, China

**Keywords:** growth cone, mammalian target of rapamycin, microglia, mitochondria, neural regeneration, oligodendrocyte, optic nerve regeneration, phosphatase and tensin homolog, phosphoinositide 3-kinase, synaptogenesis

## Abstract

Recent studies have found that the suppression of phosphatase and tensin homolog is one of the most effective single-gene approaches for promoting optic nerve regeneration. This effect is primarily mediated through the activation of the protein kinase B/phosphoinositide 3-kinase/mammalian target of rapamycin signaling pathway. The purpose of this article is to elucidate how the downregulation of phosphatase and tensin homolog is involved in each key phase of optic nerve regeneration and to summarize the potential targets for therapeutic interventions in this process. Optic nerve regeneration progresses through five phases: stress response, growth navigation, nerve regeneration, synaptic reconstruction, and remyelination. During the stress response phase, the suppression of phosphatase and tensin homolog enhances the survival of retinal ganglion cells and promotes the proliferation of microglia. In the nerve regeneration phase, reduced levels of phosphatase and tensin homolog facilitate mitochondrial transport, while inhibition of the phosphatase and tensin homolog-L isoform specifically promotes mitophagy. During the synaptic reconstruction phase, the deletion of phosphatase and tensin homolog modulates the synthesis of axon extension-related proteins and stabilizes microglial microtubules, thereby accelerating the clearance of damaged synapses and the formation of new ones. During the remyelination phase, the knockout of phosphatase and tensin homolog promotes the proliferation of oligodendrocyte progenitor cells and the differentiation of oligodendrocytes, relieving myelination obstruction. This paper also discusses current strategies and translational challenges for neuron-specific inhibition of phosphatase and tensin homolog, including off-target effects, delivery precision, and long-term safety. By integrating molecular insights with emerging bioengineering approaches, this paper provides a framework for developing targeted therapies for optic nerve regeneration and broader applications in the field of central nervous system regeneration.

## Introduction

Globally, optic nerve injury (ONI) poses a substantial public health challenge, affecting over 100 million individuals with varying degrees of severity (Sebastiano and Zack, 2021; Harada et al., 2025; Li et al., 2025). ONI can be caused by diverse optic neuropathies, including glaucoma, diabetic retinopathy, optic neuritis, ocular tumors, and trauma (Lu et al., 2020; Sebastiano and Zack, 2021). These pathologies invariably lead to irreversible degeneration of retinal ganglion cells (RGCs). Compounding this problem, optic nerve regeneration (ONR) declines progressively with age in mammals (Vanhunsel et al., 2022; Cen et al., 2023; Coleman-Belin et al., 2023). There is evidence that both extrinsic and intrinsic barriers impede nerve regeneration in the central nervous system (Canty et al., 2013; Li et al., 2022a). However, even with the removal of substantial scars, regeneration of the central nervous system remains an immense challenge. Thus, the lack of inherent regenerative capabilities becomes a contributing factor (Canty et al., 2013; Li et al., 2022a). Multiple signaling pathways have been proven to govern this inherent regenerative capacity, such as cyclic adenosine monophosphate (cAMP) (Qiu et al., 2002), suppressors of cytokine signaling 3 (SOCS3)/signal transducer and activator of transcription 3 (STAT3) (Liu et al., 2010), dual leucine zipper kinase (Saikia et al., 2022), phosphatase and tensin homolog (PTEN)/mammalian target of rapamycin (mTOR) (Park et al., 2008), and Krüppel-like factors (Hammarlund et al., 2009).

PTEN inhibition is a single-gene intervention for enhancing ONR, indicating its pivotal role in regulating intrinsic growth in the mechanisms of central nervous system regeneration. PTEN is a dual-specificity phosphatase with lipid and protein phosphatase activities (Xiong et al., 2025). It antagonizes the PI3K/AKT signaling pathway by dephosphorylating the lipid substrate phosphatidylinositol (3, 4,5)-trisphosphate (PIP3) of phosphoinositide 3-kinase (PI3K) to phosphatidylinositol (4,5)-bisphosphate (PIP2). PTEN deletion in ONR primarily activates the PI3K/AKT signaling, thereby inducing mTOR activation. The PTEN-PI3K-AKT-mTOR axis regulates different phases of ONR. There is evidence highlighting the therapeutic potential of PTEN in various optic nerve pathologies (Li et al., 2022a; Xu et al., 2023). This review positions ONR as a pioneering model for advancing research on central nervous system regeneration. The eye, as a target for gene therapy, has unique advantages including easy accessibility, compartmentalization, and relative immune privilege. These anatomical and functional advantages facilitate non-invasive intravitreal drug delivery to retinal tissues, while its immunological seclusion, enforced by the blood-retinal barrier, minimizes systemic drug dispersion (Boye et al., 2013; DiCarlo et al., 2018).

Furthermore, to observe ONR without causing procedural damage, researchers have adopted a non-invasive monitoring method that combines optical coherence tomography and visual evoked potential. This multimodal approach enhances detection of subclinical injuries and provides multi-dimensional evidence for ONR research (Trip et al., 2006). While PTEN suppression emerges as a promising therapeutic strategy for ONR, concerns have been raised about potential side effects, such as oncogenic risks, metabolic dysregulation, and cognitive impairments (Li et al., 2022a). Emerging studies indicate that selective PTEN deletion in mature neurons is highly safe and does not have notable adverse effects, thereby supporting its therapeutic viability (Gutilla et al., 2016; Gallent and Steward, 2018).

This review systematically elucidates the pivotal role of PTEN in ONR. By integrating structural analyses of PTEN with its downstream AKT/PI3K/mTOR signaling pathway, we provide mechanistic insights into how PTEN inhibits intrinsic regenerative programs. This paper comprehensively evaluates PTEN-targeting modalities and critically analyzes their therapeutic efficacy, technical limitations, and translational safety. By connecting molecular insights with translational applications, this work establishes a roadmap for overcoming barriers to central nervous system regeneration while advancing therapeutic strategies for neurodegenerative diseases, including glaucoma and traumatic optic neuropathy.

## Search Strategy

**Figure 1:**
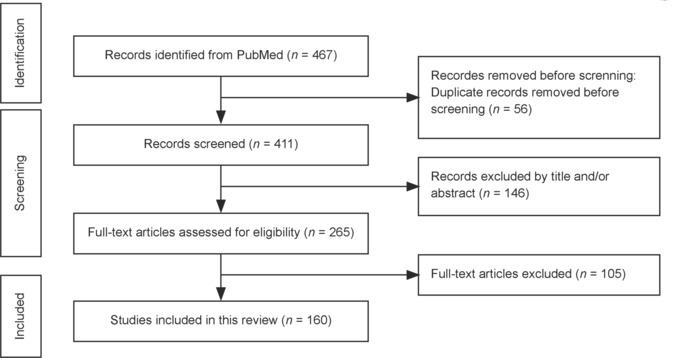
PRISMA (preferred reporting items for systematic reviews and meta-analyses) flow diagram for systematic literature reviews.

Studies were included based on the following criteria: (1) Population: Experimental animal models regardless of species, such as mice, rats, rabbits, cats, and pigs; (2) Language: Peer-reviewed English-language publications; (3) Study type: animal experiments and preclinical investigations addressing the role of PTEN in optic nerve pathophysiology, including controlled experimental designs and longitudinal observational studies; (4) Intervention: PTEN-targeting interventions (such as CRISPR/Cas9 editing, RNA silencing, viral vector-mediated modulation, and drug treatment); (5) Outcomes: mechanistic insights into the regulatory role of PTEN in optic nerve injury/disease or therapeutic efficacy of PTEN-targeting interventions. Duplicate records, redundant publications in multiple languages, and non-original research, such as commentaries and editorials, were excluded. The systematic literature screening workflow is shown in **Figure 1**.

## Phosphatase and Tensin Homolog Structure and Downstream Signal Pathway

**Figure 2:**
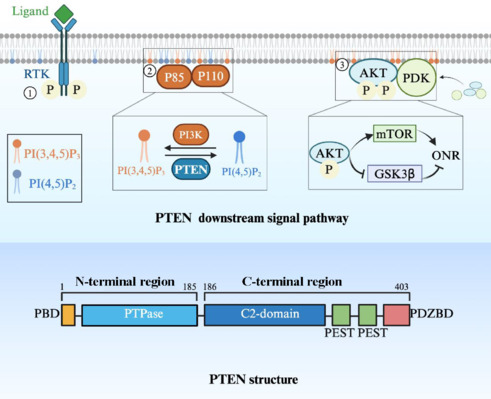
PTEN structure and downstream signaling pathway. In ①, in the ONR, RTK is autophosphorylated in response to extracellular signals, and phosphorylated RTK recruits the catalytic subunit of PI3K (p110) to the membrane by interacting with the Src homology 2 (SH2) domain on the PI3K regulatory subunit (p85). Subsequently, as shown in ②, the catalytic subunit of PI3K (p110) converts PI (4, 5) P2 to PI (3, 4, 5) P3. Finally, as shown in ③, the increase in the local concentration of PI (3, 4, 5) P3 recruits AKT and the activating kinase PDK1 to the cell membrane. PTEN, localized to the membrane via C2 domain-mediated phospholipid interactions, acts as a lipid phosphatase. Then, PTEN dephosphorylates PI (3, 4, 5) P3 to PI (4, 5) P2 through the active site pocket of the PBD to prevent the increase of local PI (3, 4, 5) P3 concentration. In this way, PTEN inhibits the recruitment of AKT and PDK1 to the membrane by PI (3, 4, 5) P3. Thus, PTEN inhibits the AKT-mediated phosphorylation of the N-terminal serine-9 of glycogen synthase kinase 3β (GSK3β-S9) and promotes the activity of GSK3β. Moreover, PTEN also inhibits the activation effect of AKT on mTORC1, both hindering ONR. PTEN is mainly composed of five domains: PTPase domain, C2 domain, PEST sequence, PDZ binding domain, and PIP2-binding domain: PBD. PTEN inhibits the PI3K-AKT-mTORC1 pathway. Created with BioRender.com. Numbers ①, ②, and ③ in the figure denote the sequence of events. AKT: Protein kinase B; GSK3β: glycogen synthase kinase 3 beta; mTOR: mammalian target of rapamycin; ONR: optic nerve regeneration; P85: PI3K regulatory subunit p85; P110: PI3K catalytic subunit p110; PBD: PIP2 binding domain; PDK: phosphoinositide-dependent kinase; PDZBD: PDZ binding domain; PI3K: phosphatidylinositol 3-kinase; PTEN: phosphatase and tensin homolog; RTK: receptor tyrosine kinase.

PTEN is mainly composed of five functional domains: the phosphatase (PTPase) domain, C2 domain, PEST sequence (targeting proteasomal degradation), PDZ binding domain, and PIP2 binding domain (PBD) (Trotman et al., 2007). Its membrane localization is mediated by C2 domain interactions with the phospholipid bilayer, where the catalytic pocket of PBD dephosphorylates PIP3 into PIP2. This dephosphorylation decreases the local concentration of PIP3, preventing the recruitment of pleckstrin homology (PH) domain-containing proteins, such as AKT and its activating kinase PDK1, to the membrane. Consequently, AKT activation is suppressed, inhibiting the PI3K/AKT/mTOR pathway (Funamoto et al., 2002). PTEN deficiency disrupts this regulatory mechanism: decreased PIP3 dephosphorylation allows PIP3 accumulation, enabling PDK1-mediated AKT phosphorylation and activation. Enhanced PI3K/AKT signaling has been shown to promote ONR (Yu et al., 2020), as detailed in the section titled “Effects of Phosphatase and Tensin Homolog on Optic Nerve Regeneration.” The antagonistic role of PTEN in the PI3K/AKT/mTOR signaling pathway is well-established (**Figure 2**; Park et al., 2008). PTEN, as a lipid phosphatase, converts PIP3 to PIP2 and suppresses the PI3K signaling pathway. PIP3 recruits AKT to the membrane, where PDK1 phosphorylates AKT at threonine-308 (T308), initiating downstream signaling (Yang et al., 2002; Manning and Cantley, 2007). Activated AKT phosphorylates the tuberous sclerosis complex (TSC1/TSC2), relieving its inhibition of mTORC1. Thus, PTEN-mediated PIP3-to-PIP2 conversion suppresses AKT/mTORC1 activation (Zhou et al., 2020a). Accordingly, PTEN deficiency activates the PI3K-AKT-mTORC1 pathway (Kim et al., 2011). Additionally, AKT phosphorylates glycogen synthase kinase-3β (GSK3β) at serine-9, suppressing its kinase activity. This phosphorylation process is critical for neuronal polarization, axon branching, and axonal growth (Guo et al., 2016).

## Effects of Phosphatase and Tensin Homolog on Optic Nerve Regeneration

**Figure 3:**
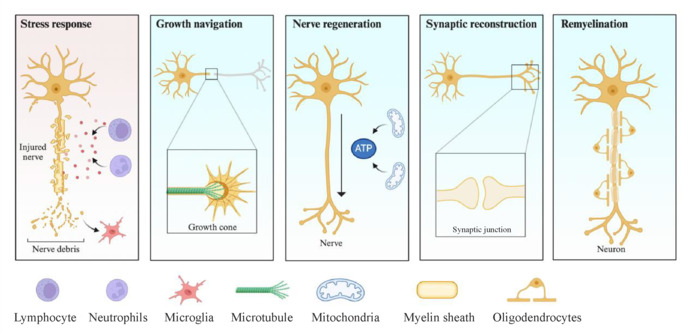
Key periods in ONR. The ONR is divided into five major periods, namely, the stress response period, growth navigation period, nerve regeneration period, synaptic reconstruction period, and remyelination period. During the stress response period, microglia transform, proliferate, and engulf neuron debris, thereby promoting ONR. During the growth navigation period, dynamically polarized microtubules drive growth cone advancement, thereby promoting axonal regeneration. During the nerve regeneration period, anterograde mitochondrial trafficking fuels ATP-dependent regeneration while suppressing ROS via PINK1/Parkin-mediated mitophagy. During the synaptic reconstruction period, axon extension promotes synapse formation. Moreover, during this period, microglia phagocytose the damaged synapse, clearing degenerative synapses to enable de novo synaptogenesis. Finally, during the remyelination period, microglia accelerate the remodeling of the myelin sheath at the site of injury by promoting oligodendrocyte precursor cell maturation. Created with BioRender.com. ATP: Adenosine triphosphate; ONR: optic nerve regeneration.

In adult mammalian species, retinal neurons usually lack the ability to regenerate after optic nerve injury. Recent research has demonstrated that deletion of the PTEN gene can trigger substantial axonal regrowth, suggesting its critical role in suppressing regenerative capacity (Fischer and Leibinger, 2012; Li et al., 2022a). ONR progresses through five successive periods: (1) stress response, (2) growth navigation, (3) nerve regeneration, (4) synaptic reconstruction, and (5) remyelination (**Figure 3**; Berry et al., 2016). Remarkably, PTEN deletion exerts a profound impact on ONR by modulating cellular and molecular mechanisms across all five stages. The following sections systematically dissect the multifaceted regulatory effects of PTEN loss, with particular emphasis on its downstream signaling pathways and interactions with key regeneration-associated factors.

**Figure 4:**
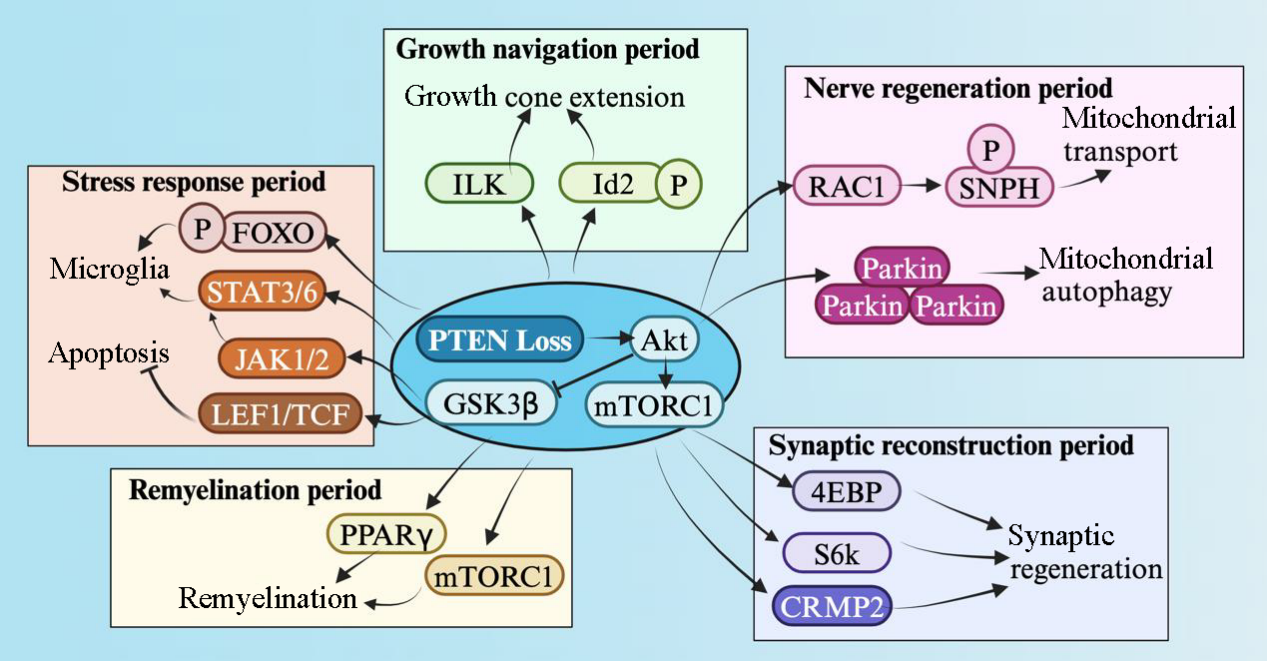
The loss of PTEN promotes ONR. Downregulation of PTEN promotes the activation of AKT. Subsequently, AKT phosphorylates TSC2, disrupting the TSC1/TSC2 complex to activate mTORC1. Concurrently, AKT phosphorylates GSK3β at Ser9, inhibiting its kinase activity to promote microtubule stabilization. Collectively, this AKT-driven dual signaling axis (mTORC1 activation and GSK3β inhibition) constitutes the core mechanism for PTEN-mediated ONR. During the stress response period, AKT activates FOXO by phosphorylation, facilitating its binding to 14-3-3 proteins. This suppresses FOXO-mediated transcription of cell cycle inhibitors, thereby enabling microglial proliferation and debris phagocytosis. GSK3β inhibition resulting from PTEN downregulation promotes β-catenin binding to LEF1/TCF to inhibit RGC apoptosis. During the late stress response period, the activation of JAK1/2 protein kinase and STAT3/6 mediated by microglia to secrete anti-inflammatory substances potentiation requires PTEN depletion. Notably, PTEN depletion also promotes neural scar formation through the upregulation of STAT3 expression. During the growth navigation period, the downregulation of PTEN enhances microtubule polymerization through GSK3β through integrin-linked kinase (ILK) to promote growth cone formation. Furthermore, PTEN downregulation also enables growth cone navigation by Id2 dephosphorylation. During the synaptic reconstruction phase, PTEN downregulation promotes axon regeneration through enhanced protein synthesis. This is achieved by relieving the inhibition on translation initiation: phosphorylation (inactivation) of the eukaryotic initiation factor 4E-BP and activation of the ribosomal protein S6K promote the assembly of the translation initiation complex. Additionally, PTEN downregulation inhibits GSK3β via phosphorylation at Ser9, thereby enhancing microtubule polymerization, which is essential for the phagocytic clearance of damaged synapses by microglia. During remyelination, enhanced PPARγ activity due to PTEN suppression promotes the anti-inflammatory secretion of microglia. These polarized microglia promote OPC proliferation. In parallel, PTEN downregulation directly drives OPC differentiation into OLs through Olig2-mediated transcriptional activation, ultimately facilitating myelin formation. Created with BioRender.com. 4E-BP: Eukaryotic translation initiation factor 4E-binding protein; AKT: protein kinase B; CRMP2: collapsin response mediator protein 2; FOXO: forkhead box O; GSK3β: glycogen synthase kinase 3 beta; Id2: inhibitor of DNA binding 2; ILK: integrin-linked kinase; JAK1/2: Janus kinase 1 and 2; LEF-1/TCF: lymphoid enhancer-binding factor 1/T cell factor; mTORC1: mammalian target of rapamycin complex 1; Parkin: Parkinson disease protein 2; PPARγ: peroxisome proliferator-activated receptor gamma; PTEN: phosphatase and tensin homolog; RAC1: ras-related C3 botulinum toxin substrate 1; S6K: ribosomal protein S6 kinase; STAT3/6: signal transducer and activator of transcription 3 and 6.

### Role of phosphatase and tensin homolog during the stress response period

Upon ONI, damaged neurons are highly vulnerable to apoptosis. The neurons first initiate intrinsic sealing mechanisms to prevent cell debris from dispersing, a process complemented by phagocytic cell activation to clear residual debris. Concurrently, glial scar formation establishes a protective barrier against neurotoxic substances. During this period, PTEN downregulation plays a critical role in ONR by enhancing microglial proliferation, phenotypic transformation, and phagocytic activity, while also promoting RGC survival post-injury (Zhou et al., 2020b; Hu et al., 2022).

During the early stress response period, reduced PTEN levels in microglia lead to AKT activation (Sarn et al., 2021). Activated AKT phosphorylates forkhead box O (FOXO) transcription factors, facilitating their binding to 14-3-3 proteins. This interaction sequesters FOXO in the cytoplasm, preventing its translocation to the nucleus. Consequently, FOXO cannot inhibit cyclin D and CDK4/6, which are key regulators of cell cycle transition from G1 to S phase (Shin et al., 2003). The inactivation of FOXO by AKT also upregulates p27 and p21 expression, further accelerating the progression of the microglial cell cycle. These combined mechanisms enhance microglial proliferation and indirectly boost their capacity to clear myelin debris through phagocytosis (Bouchard et al., 2004). In RGCs, PTEN downregulation inhibits GSK3β, preventing β-catenin phosphorylation and degradation. Accumulated β-catenin enters the nucleus (Conde-Perez et al., 2015), binds to the lymphoid enhancer-binding factor 1/T cell factor (LEF-1/TCF) transcription complex, and activates genes associated with cell growth. This process suppresses RGC apoptosis and supports neuronal survival (Wen et al., 2019). While microglia mainly mediate myelin debris clearance via proliferation and phagocytosis early in the response period, their later secretion of pro-inflammatory factors can cause secondary injury, creating obstacles for ONR. Thus, a phenotypic shift toward anti-inflammatory microglia is essential for sustained repair (Zhou et al., 2020b; Zhang et al., 2023). PTEN deficiency facilitates this shift by activating mTOR, which phosphorylates downstream targets such as p70S6 kinase (S6K) and 4E-binding proteins (4E-BPs) to enhance protein synthesis and upregulate anti-inflammatory markers such as Arg1 (Yang et al., 2023). The resulting anti-inflammatory microglia mitigate cytotoxic and oxidative stress while releasing neurotrophic factors that promote ONR (Wang et al., 2022).

### Role of phosphatase and tensin homolog in the axon pathfinding period

During the axon pathfinding period, new growth cones form at the lesion site (Verma et al., 2005). As the growth cones radially expand and microtubule dynamics increase, axonal outgrowth accelerates. Neuronal deletion of PTEN enhances axon regeneration by facilitating microtubule polymerization and elongation. Upon PTEN knockout, activated AKT specifically inactivates GSK-3β at the tip of growth cones through ILK. This inactivation prevents GSK-3β from phosphorylating the microtubule plus-end scaffolding protein adenomatous polyposis coli (APC), enabling APC to accumulate at the plus ends of microtubules. This process stabilizes microtubule polymerization and strengthens the microtubule network in growth cones, facilitating the forward advancement of growth cones and distal growth of axons (Zhou et al., 2004).

AKT also phosphorylates phosphorylated DNA binding inhibitor 2 (Id2), which is highly enriched at branch points and axon tips in regenerating axons. The phosphorylation of Id2 potentiates axonal elongation and branching. Notably, the AKT/Id2 signaling is critical for growth cone formation. The AKT/Id2 complex interacts with structural motifs in the growth cone periphery to stimulate microtubule assembly and modulate actin dynamics. This cytoskeletal reshaping propels growth cone advancement. Mechanistically, myosin-mediated cytoplasmic flow generates the driving force (Yu et al., 2011; Ko et al., 2016). However, the mechanism by which PTEN deficiency affects AKT-dependent cytoskeletal remodeling remains poorly understood, necessitating further investigation (Ko et al., 2016; Jin et al., 2018).

### Role of phosphatase and tensin homolog in the nerve regeneration period

During the nerve regeneration period, axons extend rapidly under growth cone guidance, significantly increasing energy demands. ONI might lead to mitochondrial damage, resulting in decreased energy production, reduced calcium storage capacity, and elevated reactive oxygen species (ROS), all of which hinder axonal extension (Han et al., 2020). PTEN loss in neurons enhances mitochondrial transportation to damaged optic nerve sites, meeting heightened energy and calcium buffering demands (Shlevkov et al., 2016) and promoting mitophagy to limit the production of ROS (Li et al., 2018a).

PTEN downregulation promotes AKT phosphorylation. Evidence exists that AKT phosphorylates p21-activated kinase 5 (PAK5), a brain-specific mitochondrial kinase, in neurons (Stöhr et al., 2012; Huang et al., 2021). PAK5 phosphorylates four N-terminal residues (Ser56, Ser59, Thr63, Ser64) on the mitochondrial anchoring protein syntaphilin, reducing its microtubule-binding affinity. Syntaphilin typically immobilizes mitochondria by tethering them to microtubules via its unstructured domain. Phosphorylation weakens this interaction, enabling dynamin-mediated mitochondrial transport along acetylated microtubules to injury sites (Sun et al., 2013; Huang et al., 2021).

Damaged mitochondria release ROS and occupy cellular space, requiring efficient clearance to facilitate ONR. Downregulating PTEN promotes mitophagy, a process that removes dysfunctional mitochondria, reduces ROS release, and creates space for healthy mitochondrial regeneration (Chen and Dorn, 2013). In healthy mitochondria, PINK1 is imported and rapidly degraded. By contrast, on bioenergetically impaired mitochondria, PINK1 is stabilized on the outer mitochondrial membrane (OMM), and its half-life is prolonged. PINK1 phosphorylates ubiquitin and Parkin, unlocking Parkin’s E3 ligase activity. This triggers extensive ubiquitination of OMM proteins, forming a self-amplifying signal that recruits autophagic machinery to eliminate damaged mitochondria (Gladkova et al., 2018; Wang et al., 2020b). Autophagy-related proteins then recognize ubiquitinated mitochondria and mediate their removal (Yamada et al., 2019).

PTEN-L localizes to the OMM and suppresses Parkin activation by blocking ubiquitin phosphorylation (pSer65-Ub) (Wang et al., 2018). This suppression prevents Parkin from releasing its Ub-like domain, stalling PINK1-mediated phosphorylation and hindering exposure of Parkin’s active site containing the RING2 domain. Consequently, Parkin remains inactive, disrupting the mitophagy feedback loop (Trempe et al., 2013; Li et al., 2018b; Wang et al., 2018, 2020b). However, PTEN inhibitors can reduce PTEN-L accumulation to restore mitophagy efficiency (Sivakumar et al., 2020; Zajicek et al., 2022).

The role of canonical PTEN in mitophagy remains unclear. One study shows that PTEN downregulation in neurons increases Mitofusin-2 expression through the AMPK-CREB pathway, promoting mitophagy (Li et al., 2020). Interestingly, PTEN and Mitofusin-2 localize to endoplasmic reticulum (ER)-mitochondria contact sites. PINK1-mediated mitofusin-2 phosphorylation enables Parkin recruitment and mitochondria dissociation, which are demonstrated to initiate mitophagy (Chen and Dorn, 2013). Elucidating PTEN and Mitofusin-2 interactions at these contact sites may offer novel therapeutic strategies for ONR.

### Role of phosphatase and tensin homolog during the synaptic reconstruction period

Following ONI, RGCs undergo rapid dendritic retraction and widespread synaptic loss. Therefore, synapse reconstruction is critical for subsequent repair and regeneration processes to ensure functional recovery of the optic nerve (Knafo et al., 2016). During the synaptic reconstruction period, regenerating axons extend forward while exploring target cells to form new synapses. The deletion of PTEN in RGCs enhances axonal extension through activation of the AKT pathway (Vanderplow et al., 2021).

PTEN loss activates mTORC1 signaling, stimulating downstream translation initiation factors such as 4E-BPs and S6Ks. Activated 4E-BPs release eukaryotic translation initiation factor 4E (eIF4E), enabling its interaction with cap-binding proteins. This process promotes mRNA binding to ribosomes and initiates translation (Aguilar-Valles et al., 2021). Meanwhile, activated S6Ks phosphorylate the p70S6 protein within the 40S ribosomal subunit. In its unphosphorylated state, p70S6 protein resides on the outer surface of the 40S subunit, blocking appropriate alignment with the 60S subunit. When the p70S6 protein is multiply phosphorylated, it is released from the 40S surface, accelerating ribosome assembly (Tariq et al., 2022). Multiple site phosphorylation further enhances the negative charge on the surface of the p70S6 protein, generating a localized negative electric field. A negative electric field attracts translation factors, promoting the assembly of initiation complexes and axon regeneration (Decourt et al., 2023).

Damaged synapses following ONI can impede new synapse regeneration. PTEN downregulation in microglia promotes autophagy at damaged synapses, thereby making room for subsequent synaptic regeneration (Benetatos et al., 2020). Activated GSK3β phosphorylates CRMP2 at the Thr514 residue, which induces conformational changes that impair the ability of CRMP2 to promote microtubule polymerization and stabilization (Yoshimura et al., 2005). Furthermore, GSK3β also suppresses the interactions of MAP1B and MAP2 with assembly proteins with microtubule assembly proteins, disrupting their nucleation competency, ultimately destabilizing the microtubule network (Zu et al., 2020). PTEN downregulation stabilizes microglial microtubules through GSK3β suppression, thereby enabling microglia-mediated synaptic clearance, creating space for new synapse formation.

Through this dual regulatory mechanism, enhancing axonal protein synthesis via mTORC1 activation and promoting synaptic clearance via GSK3β inhibition, PTEN plays a pivotal role in optimizing synaptic remodeling and functional recovery after ONI.

### Role of phosphatase and tensin homolog during the remyelination period

Following ONI, microglia and astrocytes are activated, releasing signaling molecules that recruit oligodendrocyte precursor cells (OPCs) from both ends of the lesion sites to demyelinated regions. These OPCs differentiate into mature oligodendrocytes to form new myelin sheaths. However, this process is often incomplete since the lesion across the optic nerve obstructs OPC migration and delays remyelination. Concurrently, reactive astrocytes secrete neurotoxins that inhibit myelin repair, while hyperactivated microglia exacerbate inflammatory damage. Collectively, these factors contribute to inefficient myelin regeneration post-injury (Osso and Hughes, 2024).

PTEN inhibition enhances OPC myelination through mTORC1 (Park et al., 2010) and peroxisome proliferator-activated receptor gamma (PPARγ) (Wang et al., 2015). In the early phase of the remyelination period, PTEN downregulation in microglia elevates the PPARγ activity, facilitating its interaction with the nuclear factor-kappa B (NF-κB) subunit p65. This interaction upregulates Arg1 and FGF2 expression while suppressing p65 activity and MKK4 kinase levels, shifting microglia toward an anti-inflammatory state (Bernhardt et al., 2022; Jiang et al., 2022). The transformed microglia express the Nrp1 receptor that binds PDGFRα on the surface of OPCs, initiating a signaling cascade where PIP3 activates AKT via ATP-dependent PIP2 phosphorylation. AKT then regulates multiple key cell cycle checkpoints to promote OPC proliferation, accelerating myelin sheath formation (Bernhardt et al., 2022).

For functional remyelination, proliferated OPCs must differentiate into myelinating oligodendrocytes (OLs). PTEN downregulation primarily promotes this process through mTORC1 (Wahl et al., 2014; Goebbels et al., 2017). In OPCs, downregulation of PTEN activates the PDK1/AKT pathway upstream of mTORC1, specifically increasing mTORC1 activity. Activated mTORC1 phosphorylates translational repressor 4E-BP to release the eIF4E (Rosenwald et al., 1993), activates p70S6K (Li et al., 2022a), and promotes c-Myc translation (Wall et al., 2008), thereby enhancing the transcription of differentiation genes and the initiation of protein translation (Chan et al., 2014; Rivera et al., 2022).

Despite PTEN knockout in RGCs enhancing OPC differentiation via the mTOR pathway, regenerated RGC axons remain unmyelinated and functionally impaired. This might be attributed to ONI-induced upregulation of G protein-coupled receptor 17 (GPR17), which can suppress the differentiation and maturation of OPC and impede myelin sheath formation (Fumagalli et al., 2015; Shao et al., 2021). Therefore, we propose that combination of GPR17 antagonists and PTEN knockout in OPCs would enhance the remyelination process after ONI (Wang et al., 2020a).

## Role of Phosphatase and Tensin Homolog in Optic Nerve Disorders

Optic nerve disorders encompass conditions characterized by damage to the optic nerve resulting from various pathological processes (Hall et al., 2025; Norte-Muñoz et al., 2025; Wang et al., 2025). Mechanistically, PTEN acts as a critical intrinsic inhibitor of axon regeneration in the central nervous system, where its deletion in RGCs represents one of the most effective single-gene manipulation strategies to promote ONR after ONI (Tang et al., 2022). Notably, PTEN modulation in RGCs demonstrates therapeutic potential for repairing optic nerve damage in multiple optic neuropathies (Jacobi et al., 2022).

### Glaucoma

Glaucoma encompasses progressive optic neuropathies characterized by RGC degeneration and optic nerve head remodeling. Classified by the anatomical configuration of the anterior chamber angle, it manifests as two major forms: primary open-angle glaucoma and primary angle-closure glaucoma (Weinreb et al., 2014). To counteract these degenerative processes, PTEN downregulation has emerged as a critical strategy to promote ONR in glaucoma. Under glaucomatous stress, reduced PTEN activity permits Akt-dependent mTORC1 activation. This kinase phosphorylates S6K to enhance protein translation through ribosomal protein S6 phosphorylation, supplying materials for axonal repair; its contribution to ribosome biogenesis requires further investigation (Li et al., 2022a). In addition, reduced PTEN expression specifically upregulates Annexin A2 and membrane palmitoylated protein 1. Annexin A2, a calcium-dependent phospholipid-binding protein, binds with cell surface receptors to activate the plasminogen system. This enzymatic process degrades extracellular matrix barriers and creates a supportive microenvironment for axonal regrowth. Annexin A2 can also interact with integrins to activate ILK, facilitating RGC polarization and growth cone guidance (Wei et al., 2023). Concurrently, another protein upregulated following PTEN inhibition, membrane palmitoylated protein 1, can enhance growth factor responsiveness and synergistically promote axon extension (Lascaratos et al., 2017).

### Traumatic optic neuropathy

Direct or indirect injury to the optic nerve triggers traumatic optic neuropathy, typically presenting as significant loss of RGCs and their axonal fibers. This neurodegeneration results in partial visual deficits or irreversible vision loss (Au and Ma, 2022). However, PTEN downregulation can promote ONR in traumatic optic neuropathy by regulating mitochondrial transport and reducing inflammation (Steinsapir and Goldberg, 2011; Xie et al., 2022). Mechanistically, the absence of PTEN potently activates the AKT/PAK5 signaling pathway, thereby uncoupling mitochondria from microtubules (Steinsapir, 1999; Wladis et al., 2021; Han et al., 2023). This liberation enables mitochondrial trafficking to injury sites to meet energy demands. In addition, PTEN deficiency can also induce mTORC1-mediated microglia polarization, upregulating key anti-inflammatory genes (Sun et al., 2021). This concurrently inhibits NF-κB nuclear translocation and reduces pro-inflammatory cytokine release, thereby mitigating neuroinflammation (Baechler et al., 2019; Li et al., 2022b).

### Optic neuritis

Optic neuritis is an inflammatory optic neuropathy, often presenting as an inflammatory demyelinating disease of the optic nerve. As a common initial manifestation of multiple sclerosis (MS), optic neuritis shares core pathogenic mechanisms with MS (Saitakis and Chwalisz, 2022; Kraker and Chen, 2023).

Th22 cells, a novel CD4^+^ T cell subset involved in the development of MS, are elevated in optic neuritis. These cells secrete interleukin-22, which activates NF-κB to suppress PTEN expression in T cells. PTEN loss in T cells activates AKT while suppressing FOXO1, a transcription factor that promotes Ikaros expression (Toosy et al., 2014). Ikaros, in turn, upregulates FOXP3, a key regulator of CD4^+^CD25^+^FOXP3^+^ regulatory T cells (Tregs) (Benard-Seguin and Costello, 2023). FOXP3 deficiency disrupts Treg-mediated suppression of effector T cells, exacerbating inflammation (Kang et al., 2021; Saitakis and Chwalisz, 2022). It can be expected that PTEN loss in T cells exacerbates the inflammatory response in optic neuritis by driving excessive T cell proliferation and cytokine release.

The regulatory effects of PTEN exhibit cell-specific and spatiotemporal complexity. While PTEN inhibition in T cells worsens inflammation, PTEN downregulation in microglia activates mTORC1 by releasing Akt suppression, driving anti-inflammatory polarization. This dichotomy phenomenon suggests that the regulatory effects of PTEN have significant cell-specific and spatiotemporal dependence. Therefore, the intervention strategies for PTEN need to precisely target the appropriate cell type and pathological stage to optimize therapeutic outcomes (Cossu et al., 2021).

Matrine, a marine-derived alkaloid, alleviates neuroinflammation and preserves myelin integrity in optic neuritis by inhibiting PI3K/AKT/mTOR signaling in microglia and astrocytes (Song et al., 2022; Kraker and Chen, 2023). Interestingly, PTEN ablation in RGCs enhances AKT phosphorylation, promoting neuronal survival in neurodegenerative models. We hypothesize that combinatorial targeting, suppressing glial AKT activation via matrine while enhancing neuronal AKT signaling through RGC-specific PTEN inhibition, may achieve synergistic neuroprotection. This cell type-specific dual modulation strategy warrants further investigation for optic neuritis therapy.

### Papilledema

Papilledema, defined as optic disc edema resulting from elevated intracranial pressure, is a critical complication of intracranial hypertension, often leading to vision loss. Its pathophysiology depends on the interplay of three key pressures: arterial pressure, intracranial pressure, and intraocular pressure (Lee and Wall, 2012). The pathogenic cascade initiates when intracranial pressure exceeds intraocular pressure, reducing optic disc perfusion pressure. The reduction of optic disc perfusion pressure impairs axonal transport, causing axoplasmic stasis at the rigid lamina cribrosa, leading to optic nerve damage (Schirmer and Hedges, 2007; Raoof and Hoffmann, 2021).

However, the downregulation of PTEN seems to alleviate optic nerve damage induced by papilledema and promote the regeneration of damaged optic nerves. Recent studies have shown that under ischemic conditions, AKT phosphorylation can protect RGCs and promote the synthesis of RGC-related proteins, thereby facilitating the repair of damaged optic nerves (Lee et al., 2022; Zhang et al., 2024). Downregulation of PTEN promotes the phosphorylation of AKT. Activated AKT inhibits the activity of pro-apoptotic protein Bad by phosphorylating it. Additionally, AKT can phosphorylate the tumor suppressor TSC2, thereby relieving the inhibition of small GTPase Rheb by TSC2 (Nishinaka et al., 2018). Activated Rheb promotes the activation of mTORC1, which phosphorylates S6K. The activation of S6K further enhances the transcription of rRNA and the biogenesis of ribosomes, thereby promoting protein synthesis. mTORC1 also phosphorylates 4E-BP1 (eIF4E-binding protein), thereby releasing eIF4E from 4E-BP1 to promote protein synthesis (Weidman et al., 2020). Therefore, downregulation of PTEN improves RGC function by enhancing AKT phosphorylation, thereby promoting ONR.

### Optic nerve atrophy

Optic atrophy refers to a morphological change characterized by the thinning of the optic nerve due to pathological changes in the RGCs and their axons (Chun and Rizzo, 2017; Charoenkijkajorn et al., 2022). Optic neuropathy can be divided into two types: primary and secondary, among which the most common are hereditary optic neuropathy, primary optic atrophy (mainly dominant optic atrophy), and Leber’s hereditary optic neuropathy.

The downregulating PTEN alleviates optic nerve damage caused by optic atrophy by facilitating mitophagy and transport. The downregulation of PTEN in damaged cells can enhance AKT phosphorylation and activation, which in turn promotes PAK5 activation. Activated PAK5 facilitates the separation of mitochondria from microtubules by interacting with the four phosphorylation sites of Syntaphilin (Knafo et al., 2016). This microtubular detachment promotes mitochondrial trafficking, thereby optimizing bioenergetic support for the ONR. In addition, PINK1 stabilization on depolarized mitochondria triggers widespread ubiquitination, promoting mitophagy, a process enhanced by PTEN downregulation (Huang et al., 2021). However, in the case of dominant optic atrophy caused by mutations in OPA1, studies have shown that AKT phosphorylation promotes the cleavage of OPA1 (Wang et al., 2020b; Chen et al., 2022). Activated AKT phosphorylates the mitochondrial dynamin DNM1L, and the activated DNM1L directly interacts with OPA1 to promote its cleavage. Then, targeted suppression of PTEN in RGCs under mitochondrial stress potentiates mitophagy, while PTEN maintenance in RGCs under physiological conditions suppresses OPA1 proteolysis, which may establish an endogenous neuroprotective pathway in RGCs against bioenergetic failure. This hypothesis still needs to be validated through experiments (Iskandar et al., 2024).

### Diabetic optic neuropathy

Diabetic optic neuropathy (DON), as one of the complications of diabetes, has not yet been fully elucidated regarding its pathogenesis (Selvarajah et al., 2019). DON can cause disruptions in the axon-glial connections of the optic nerve and demyelination-induced axonal degeneration, resulting in optic nerve damage. In patients with diabetes mellitus, elevated blood sugar levels lead to the activation of the polyol pathway (Hua et al., 2019). Excess glucose undergoes aldose reductase-mediated conversion to sorbitol. Accumulation of this metabolite results in a decrease in intracellular levels of myo-inositol. Myo-inositol is crucial for maintaining the normal function of glial cells and regulating osmotic balance via Na^+^/ATPase modulation, and its deficiency weakens the support that glial cells provide to axons. This decline in supportive capability makes the connection between axons and glial cells unstable, resulting in axo-glial dysjunction (Kamijo et al., 1993). As the degree of separation between axons and glial cells progressively increases, the energy supply to axons becomes insufficient, leading to demyelination. This degenerative process compromises neuronal survival and functionality, thereby impairing optic nerve conduction efficiency and resulting in progressive visual field deficits (Li et al., 2021). The downregulation of PTEN enhances AKT phosphorylation and inhibits the polyol pathway, thereby reducing optic nerve damage caused by DON and promoting ONR (Tan et al., 2021). The downregulation of PTEN activates AKT phosphorylation, and the activated AKT inhibits the activity of aldose reductase by phosphorylating it. Additionally, AKT can also phosphorylate and inhibit the activity of the transcription factor NF-κB, thereby preventing NF-κB from binding to the promoter region of the aldose reductase gene to promote its transcription. Paradoxically, in diabetic hyperglycemia, activated aldose reductase reciprocally inhibits AKT phosphorylation, forming a pathogenic feedback loop (Tarkkonen et al., 2023). Aldose reductase activation depletes intracellular NADPH, disrupting redox homeostasis and inhibiting AKT phosphorylation. In addition, aldose reductase can exacerbate oxidative stress within the cell, increasing the levels of reactive oxygen species, which directly damage the structure and function of AKT (Chen et al., 2023). PTEN downregulation enhances AKT phosphorylation, disrupting the aldose reductase-AKT reciprocal inhibition loop and suppressing aldose reductase activity (Ali et al., 2019). Consequently, this intervention blocks the polyol pathway, reduces optic nerve damage, and promotes optic nerve regeneration (Tang et al., 2022).

## Potential Treatment Strategies for Targeting the Phosphatase and Tensin Homolog Gene

There are many strategies to inhibit the function of PTEN, such as bisperoxovanadium (bpV) (Kurimoto et al., 2010). However, bpV compounds block various phosphatases, such as leukocyte common antigen-related phosphatase (LAR) and T-cell protein tyrosine phosphatase (Scrivens et al., 2003; Fisher et al., 2011). Notably, LAR has been identified as a functional receptor of scar-derived inhibitors in mediating axonal growth (Fisher et al., 2011). Hence, the imperative lies in developing a pharmacological approach that achieves efficient modulation of PTEN while ensuring specificity.

Based on the distinct mechanisms and modes, the existing regulatory strategies for PTEN can be categorized into functional regulation approaches targeting PTEN activity and delivery systems for precision therapeutic intervention. Functional regulation strategies, including PTEN antagonistic peptides (PAPs), CRISPR-dCas9 epigenetic editing, RNA interference, and coordinated strategies, exhibit advantages such as high specificity, safety, and efficiency. Delivery systems, such as adeno-associated viruses (AAVs) and nanoparticles, demonstrate controllability and the ability to facilitate drug absorption. In **Additional Table 1**, we discussed the advantages and limitations of each methodology and clearly outlined the regenerative effects of these regulatory approaches in **Additional Table 2**.

**Additional Table 1:** Functional regulation strategies and delivery systems for phosphatase and tensin homolog modulation: strengths and weaknesses

Therapy	Strength	Weakness and prospect	Reference
PTEN antagonist peptides (PAPs)	(1) High specificity: It targets the structural domain of PTEN. (2) High safety: It suppresses PTEN rather than knockout. (3) Its effect is stronger than bpV.	(1) The promoting effect of PAP on ONR was not as significant as deleting PTEN. (2) Its effect might be enhanced by loading it onto nanoparticles or other carriers.	Ohtake et al., 2014
CRISPR-dCas9	(1) Simple and efficient: The design and implementation are easy. (2) Low cost: It has low cost with simple equipment and operation. (3) High safety: It achieves reversible inhibition.	(1) It cannot be applied to all eukaryotic cells. (2) This repair process is prone to mutations such as insertions and deletions.	Ahmar et al., 2023; Saito et al., 2023
SdRNA	(1) Penetrates the cell membrane autonomously: It does not rely on delivery vehicles. (2) Chemical modification can heighten stability, prolong duration, reduce immunogenicity and thus improve safety.	(1) Chemical modification raises costs. (2) Optimizing the target gene sequences for diverse species necessitates further labor. (3) It solely applies to localized ailments such as ocular injuries.	Woller et al., 2022
Adeno-associated virus (AAV)	(1) High efficiency: Cre enzyme expresses over 90% RGCs following AAV injection. (2) Controllable: The gene is only removed when transited from the floxed status to null status after AAV-Cre injection. (3) rAAV-retro, AAVrg, and Y444F-AAV exhibit high retrograde transduction efficiency with prominent selectivity.	(1) Knockout efficiency is not 100% comprehensive. (2) AAV, acting as an exogenous expression vector, can potentially trigger nonspecific immunological reactions.	Huang et al., 2018; Park et al., 2008; Metcalfe et al., 2022; Stewart et al., 2023
Nano-particle	(1) Various functions: It can carry diverse kinds of medications. (2) Controlled release of medications: without duplicate administration frequently. (3) Absorb efficiently: further facilitating pharmacological impact. (4) PLGA degrades harmlessly in the body and has relatively small toxic side effects.	(1) It entails intricate processing, such as electrochemical etching and fracturing. (2) Loading medications onto nanoparticles results in some wastage.	Kim et al., 2016; Zuidema et al., 2020
Coordinated strategy	(1) Powerful regeneration: Concurrent multi-mechanism synergism renders effects stronger than singular treatment methods. (2) It can maximize RGC survival and regeneration whilst minimizing medication dosages.	(1) Developing selective and safe therapeutic approaches grounded upon multi-target pharmacological strategies presents considerable difficulties. (2) Execution is intricate, necessitating superabundant steps. (3) Optimizing relative dosages amongst diverse medications still requires further exploration.	Kurimoto et al., 2010; Sun et al., 2011; Bei et al., 2016; Li et al., 2017

This table outlines PTEN modulation strategies. These strategies are categorized into two main types: functional regulation approaches, such as PAPs, CRISPR-dCas9, and SdRNA, which directly target PTEN activity, and delivery systems like AAV, nanoparticles, and coordinated strategies, which optimize therapeutic delivery. Functional strategies offer specificity. For example, PAPs target PTEN domains. However, they face limitations. CRISPR has off-target effects, and SdRNA has scalability issues. Delivery systems enhance precision. For instance, AAV-mediated delivery can be used for cell-specific knockout. Besides, they need to be optimized for safety, considering immune responses, and for efficiency, particularly in nanoparticle fabrication. AAV and SdRNA show promise for ocular applications due to localized delivery. Coordinated strategies, like PTEN/SOCS3 co-deletion, achieve synergistic regeneration. But they demand rigorous safety validation. The table underscores PTEN modulation as a viable regenerative approach, highlighting combinatorial methods to address challenges, thereby advancing translational research for optic nerve and central nervous system injuries. AAV: Adeno-associated virus; bpV: bisperoxovanadium; CRISPR-dCas9: clustered regularly interspaced short palindromic repeats-dead Cas9; PAPs: PTEN antagonist peptides; PLGA: polylactic-co-glycolic acid; PTEN: phosphatase and tensin homolog; SdRNA: self-delivering RNA.

**Additional Table 2:** Regenerative effects of phosphatase and tensin homolog functional regulation strategies and delivery systems

Therapy	Model	Approach	Major findings (compared with controls)	Reference
PAP1–4	DRG from C57BL/6 adult mice	The DRG cultured *in vitro* was treated with PAP1–4 to inhibit PTEN.	The growth length of DRG was 1.7 to 2 times compared with controls after 5 d.	Ohtake et al., 2014
PAP1–4	CGN from C57BL/6 adult mice	The CGN cultured *in vitro* was treated with PAP1–4 to inhibit PTEN.	The growth length of CGN was 1.6 to 2 times after 7 d.	Ohtake et al., 2014
PAP2 PAP4	Adult C57BL/6 mice	(1) Dorsal hemisection injury in the spinal cord of mice. (2) PAP2 or PAP4 was injected twice daily for 14 d to inhibit PTEN.	(1) The number of 5-HT positive axons within the 5-7 mm beyond the injury site was twice that of the controls. (2) Improvement in locomotor function scoring, grid walk testing, and footprinting analysis.	Ohtake et al., 2014
CRISPR-dCas9	PC-12 cell derived from neural crest	(1) NGF was injected to induce differentiation for 48 h. (2) The dCas9-KRAB was delivered into PC- 12 cells for further 24 h to inhibit PTEN.	The axon length was twice that of the controls after 4 d.	Moses et al., 2020
CRISPR-dCas9	hMPCs	The dCas9-KRAB ws delivered into hMPCs for 48 h to inhibit PTEN.	PTEN mRNA expression was decreased to 10% of control levels.	Moses et al., 2020
CRISPR-dCas9	hNSCs	The dCas9-KRAB was delivered into hNSCs for 48 h to inhibit PTEN.	PTEN mRNA expression was decreased to 20% of control levels.	Moses et al., 2020
SdRNA	Hippocampal and cortical neurons in adult female SD rats	(1) Neurons were isolated and seeded onto a previously established microfluidic chip. (2) Fluorescent tracer was immediately applied to the axon terminal region after the administration of sdRNA.	SdRNA was directly absorbed by primary neurons, expressed continuously, and was capable of translocation via the axon.	Woller et al., 2022
SdRNA	Adult female SD rats weighting 200-220 g	The sdRNA was intravitreally injected immediately to inhibit PTEN, while optic nerve was injured.	2 wk after injury: (1) PTEN expression was downregulated by more than 50%. (2) The maximum regenerated axon length was increased 1-fold. (3) Within 1-2 mm of the injury site, the number of regenerating axons was increased 4-fold.	Woller et al., 2022
siRNA	Adult male CD-1 12-wk-old mouse models of induced diabetes mellitus	The siRNA was intravitreally injected immediately to inhibit PTEN, while left sciatic nerve is injured.	4 wk after injury: (1) The CMAP amplitude was increased by 1-fold. (2) Motor and sensory nerve conduction velocity was increased. (3) The number of myelinated axons was increased by 1.3-fold. (4) The mechanical and thermal sensitivity thresholds were recovered.	Singh et al., 2014
IncRNA	Adult female SD mice weighting 220-230 g	The lncRNA was intravitreally injected immediately to inhibit PTEN, while sciatic nerve was injured.	18 d after injury: (1) Proliferation and migration of Schwann cell were increased by 1-fold. (2) The distances of regenerating spinal cord were increased by 1-fold.	Ma et al., 2020
AAV2-Cre	Adult C57BL/6 mice	AAV-Cre was intravitreally injected to delete PTEN, 2 wk before optic nerve injury induction.	2 wk after injury: (1) The survival rate of RGC was increased from 20% in controls to 45%. (2) Within the surviving cells, 8% to 10% of axons exceeded the 0.5 mm mark beyond the site of injury. 4 wk after injury: (3) Certain axons extended into the optic chiasm.	Park et al., 2008
rAAV-retro-Cre	Adult mice aged 8-12 wk	The rAAV-retro was intravitreally injected into the cervical spinal cord segment C5 to delete PTEN.	3 wk after injury: (1) High retrograde transduction efficiency. (2) High retrograde transduction selectivity: transducing only projection neuron of certain site.	Metcalfe et al., 2022
Y444F mutant AAV-shRNA	Adult SD mice aged 8 wk	Mutant AAV-shRNA was intravitreally injected to inhibit PTEN 4 wk before optic nerve injury induction.	(1) The retinal infection efficiency of Y444F mutant AAV was increased by 1-fold. 6 wk after injury: (2) The RGC survival rate after injury was improved to 19%. (3) Axons regenerated near the optic chiasm.	Huang et al., 2018
AAVrg-hSynl-Cre	Adult C57BL/6 mice	(1) Spinal cord was injured by crushing the T8 segment of the spinal cord. (2) AAVrg was immediately administered by lumbar injection to delete PTEN.	(1) HSyn1 promoter allowed Cre recombinase to be specifically expressed in neuron. 9 wk after injury: (2) The proportion of 33-tubulin-positive axons in the lesion area increased 2-fold to 30%, up from 15% in the control group.	Stewart et al., 2023
PLGA-pSiNPs	DRG from infant C57BL/6 mice	The PLGA-pSiNPs-bpV was injected into DRG *in vitro* to inhibit PTEN.	(1) The mass loading of bpV was 16%. (2) The PLGA-pSiNPs-bpV released 38% of bpV within the first 2 d and nearly half of bpV within 1 d. 7 d after injury: (3) The neurite length reached 4500 μm.	Zuidema et al., 2020
MSNs	DRG from SD mice aged 6-8 wk	The MSNs-bpV was injected into DRG *in vitro* to inhibit PTEN.	(1) The content of bpV was 27 μg/mg. (2) The bpV was gradually released in a slow and controlled manner within 10 d. 5 d after injury: (3) The neurite lengths increased to 3100 μm and 2700 μm, respectively, in the groups treated with 10 ng/mL and 20 ng/mL MSNs-BpV.	Kim et al., 2016
Co-deIetion SOCS3 and PTEN	Thy-1-YFP transgenic mice	(1) Administer intraperitoneal injections of tamoxifen (1 mg in 100 μL of corn oil) for 5 d to inhibit SOCS3, along with intravitreal injections of AAV to inhibit PTEN. (2) Optic nerve was injured after 2 wk.	(1) Co-deletion activated mTOR and STAT3 signal pathways in a synergistic manner. 2 wk after injury: (1) In the SOCS3 and PTEN double-knockout group and the PTEN single-knockout group, the axons of surviving RGCs appeared intact in the retina after injury. In contrast, the SOCS3 single-knockout group exhibited signs of axon degeneration. (2) The rate of aberrant axon regeneration in the retina was lower in the SOCS3 and PTEN double-knockout group. 2 mon after injury: After optic nerve injury, the survival rate of RGCs in the SOCS3 and PTEN double-knockout group was 85%, compared to 25% in the control group.	Mak et al., 2020
Co-deletion SOCS3 and PTEN	C57BL/6J mice aged 8-12 wk	(1) AAV2-Cre was intravitreally injected to delete both the PTEN and SOCS3. (2) Optic nerve was injured after 2 wk.	(1) Co-deletion activated both mTOR and STAT3 signal pathways in a synergistic manner. 4 wk after injury: (2) At 2 mm from the injury site, the number of regenerating axons was ten times greater than that in the single PTEN deletion group. (3) Successful synaptic connections within the optic chiasm and subcortical visual areas were obtained	Sun et al., 2011; Bei et al., 2016
PTEN deletion and injecting Zn2+chelators	Adult C57BL/6 mice	(1) AAV2-Cre was intravitreally injected to delete PTEN. (2) Zn^2+^ chelators were administered via intraocular injection on the day of optic nerve injury induction and again 4 d post-surgery.	2 wk after injury: RGC survival rate was increased to 46%, while single PTEN deletion was 35%.	Li et al., 2017
PTEN deletion with Zymosan and cAMP application	Adult C57BL/6 mice	(1) AAV2-Cre was intravitreally injected to delete PTEN. (2) Optic nerve was injured after 2 wk, concurrently with intraocular injection of CPT-cAMP and Zymosan.	2 wk after injury: (1) The number of regenerating axons extending beyond 3 mm from the injury site exceeded 100, which is 10 times greater than that in the single PTEN deletion group. 6 wk after injury: (2) Regenerating axons were observed projecting beyond the optic chiasm and extending into the contralateral thalamus.	Kurimoto et al., 2010
PTEN inhibtion with CNTF and cAMP application	Adult C57BL/6 mice	(1) AAV2-shRNA and AAV-CNTF were intravitreally injected to inhibit PTEN and provide CNTF. (2) Optic nerve was injured after 2 wk, concurrently with intraocular injection of CPT-cAMP and Zymosan.	2 wk after injury: (1) Some regenerated axons projected beyond the optic chiasm and entered the contralateral optic nerve. (2) Some regenerated axons entered the visual target area, forming complex dendritic arbors and establishing synaptic connections.	Yungher et al., 2015
PTEN inhibtion and salmon fibrin application	Adult female C57BL/6 mice	(1) AAV2-shRNA was injected in motor cortex to inhibit PTEN. (2) C6 segment of the spinal cord was injured after 5-7 d, concurrently with injection of salmon fibrin.	3 wk after injury: At 1 mm distal to the injury site, the number of BDA-labeled regenerating CST axons was twice that of the controls. 10 wk after injury: Forelimb grasping function recovery, assessed by the percentage of successful food pellet grasps, was approximately 48%, which is 2 to 3 times greater than that observed in the control group.	Lewandowski and Steward, 2014

This table lists six PTEN-targeting methods, including functional regulation techniques such as PAPs, sdRNA, AAV, CRISPR, and combinatorial strategies. It validates PTEN modulation as a viable regenerative approach, emphasizing combinatorial and cell-specific strategies to enhance efficacy and safety for optic nerve and central nervous system injuries. AAV: Adeno-associated Virus; BDA: biotin-conjugated dextran amine; bpV: bisperoxovanadium; CMAP: compound muscle action potential; CNTF: ciliary neurotrophic factor; cAMP: cyclic adenosine monophosphate; CGN: cerebellar granule neuron; CST: corticospinal tract; DRG: dorsal root ganglion; hMPCs: human mesenchymal precursor cells; hNSCs: human neural stem cells; lncRNA: long non-coding RNA; MSNs: mesoporous silica nanoparticles; PTEN: phosphatase and tensin homolog; PAP: PTEN antagonist peptides; pSiNPs: porous silicon nanoparticles; RGC: retinal ganglion cells; SOCS3: suppressors of cytokine signaling 3; SD mice: sprague-dawley mice; SdRNA: self-delivering RNA; SiRNA: small interfering RNA; 33- Tub: β-III tubulin.

### Phosphatase and tensin homolog antagonistic peptides

Ohtake et al. (2014) devised four distinct PAPs, namely PAP1–4, targeting different functional domains of PTEN. These peptides specifically targeted the PIP2 domain, ATP binding domain type A, ATP binding domain type B, and PDZ domain. This groundbreaking study emphasizes the potential of PAPs, targeting different functional domains of PTEN, to enhance ONR. However, this technique is only in the animal experimental stage, and its application in specific optic nerve diseases still needs further confirmation.

### CRISPR genome editing methods

In prokaryotes, there exists an ancient immune system known as CRISPR-Cas, which can defend against invasive genetic elements through targeted interference with foreign nucleic acids. In light of this property of CRISPR-Cas, scientists have developed a series of powerful gene-editing tools, among which the most renowned is CRISPR-Cas9 (Ahmar et al., 2023). Moses et al. (2020) used a dead Cas9 (dCas9) that cannot cleave DNA and fused it with the Krüppel-associated box (transcriptional repressor KRAB’s domain) to form a complex. With the guide RNAs, they specifically targeted this complex to the regulatory region of the PTEN gene. Given that the Krüppel-associated box domain can recruit chromatin-modifying enzymes, it induced heterochromatin formation in this region and then achieved regulation of *PTEN* expression. While CRISPR-Cas has not directly targeted PTEN for use in optic nerve diseases, it has been used in the treatment of CEP290-associated hereditary retinal degeneration (Sharma et al., 2021). CEP290-related hereditary retinal degeneration is an eye disease caused by mutations in the CEP290 gene, primarily affecting the photoreceptor cells in the retina, leading to severe vision loss. The most common type of mutation in the CEP290 gene associated with this disease is the IVS26 variant, which is a mutation in the 26th intron, leading to the abnormality of the CEP290 protein. The CEP290 protein is essential for the structural integrity of the photoreceptor cilia in photoreceptor cells; its deficiency leads to dysfunction of both rod and cone cells, ultimately leading to vision loss. By applying the CRISPR-Cas system to repair the IVS26 mutation, it restores normal CEP290 protein function, symptomatically ameliorating symptoms of CEP290-associated hereditary retinal degeneration (Pierce et al., 2024). The CRISPR-based EDIT-101 therapy targeting IVS26 mutations in the CEP290 gene for treating inherited retinal degeneration has achieved significant progress in clinical trials, leveraging the tropism of AAV5 for retinal cells to mediate CRISPR-Cas9 delivery for precise gene mutation repair. In the Phase I/II trial involving 14 adult and pediatric patients receiving a single subretinal injection, the results demonstrated favorable safety with no clinically significant adverse reactions observed. Among these patients, 43% of patients exhibited significant improvement in cone photoreceptor light sensitivity (a decrease of ≥ 0.6 log cd·s·m^–2^ compared to baseline after treatment), with 29% achieving marked enhancement in visual acuity (an improvement of 0.3 logMAR units compared to baseline after treatment). Although limited by a small sample size, this trial marked the first-in-human validation of CRISPR gene editing feasibility and opened new possibilities for leveraging this technology to downregulate PTEN for promoting ONR.

### RNA interference technology

In 2023, the Nobel Prize in Physiology or Medicine was bestowed upon messenger RNA (mRNA) technology. Circular RNA (circRNA) is a covalently closed circular structure, which protects it from degradation by nucleases, thus endowing it with superior stability and a longer lifespan. Furthermore, Wesselhoeft et al. (2018) demonstrate that engineered circRNA can be stably and efficiently expressed in eukaryotic cells. To investigate further, Cao et al. (2022) have uncovered that circRNA changes after peripheral axon injury are associated with metabolism and PTEN-related pathways. Self-delivering RNA (sdRNA) is a modified, small, double-stranded inhibitory RNA where the guiding strand, using cholesterol moieties at both ends, achieves cellular entry without the need for a carrier and also protects the sdRNA. Some researchers have designed and synthesized 20 siRNA sequences targeting rat PTEN, among which one sequence, BA-434, exhibited optimal performance (Hatchi et al., 2021; Woller et al., 2022). This technology has been proven effective in rat models of optic nerve injury; however, its safety in human applications and whether it is effective in specific optic nerve disorders still lack clinical trial validation.

### Associated viruses

AAV2 has been employed without notable adverse effects in clinical trials targeting both ocular and non-ocular ailments as one of the most effective vectors for transducing adult RGCs through intravitreal injection (Kotterman and Schaffer, 2014). Park et al. employed AAV to deliver Cre recombinase (AAV-Cre) into the eye of adult mice, thereby inducing conditional knockout of the PTEN gene in mature RGCs (Park et al., 2008). Furthermore, some scholars sought to assess the potential of AAV2 vectors incorporating RNA interference in suppressing the expression of PTEN (Huang et al., 2018). In addition, it has been discovered that the combination of AAV-shRNA with salmon fibrinogen administration can exert a more pronounced effect (Lewandowski and Steward, 2014). However, since it is typically recommended to administer AAV injections at least two weeks in advance to achieve therapeutic levels of gene expression in the retina, this requirement hinders efficient implementation in clinical trials (Park et al., 2008). Fortunately, Stewart et al. (2023) applied AAVrg, Metcalfe et al. (2022) applied rAAV-retro, and Liu et al. (2010, 2021) applied mutant Y444F-AAV (Huang et al., 2018). These AAV vectors incorporate specially engineered envelope proteins that significantly enhance the retrograde transport capability and reduce the time required for PTEN knockout. Stewart et al. (2023) further applied the AAVrg-hSyn1-Cre recombinant virus. By taking advantage of the specific design of the virus, it ensured exclusive expression in neurons. This, in turn, enhanced safety. Using these optimized AAVs, even when administered immediately after optic nerve injury, can produce favorable effects in promoting ONR compared with traditional methods.

In optic nerve crush, AAV (adeno-associated virus) is used as a vector to deliver Cre recombinase. By injecting AAV-Cre into the eye, the PTEN gene can be effectively knocked out in specific cells. After the injection of AAV-Cre, the *PTEN* gene is deleted, thereby activating the signaling pathways associated with ONR. Studies have shown that the loss of PTEN in mice significantly increases the survival rate of RGCs after optic nerve crush (Arcuri et al., 2021; Liu et al., 2021). By comparing with the control group (using AAV-PLAP), the experimental group showed a significantly higher number of surviving RGCs after a certain period (such as 7 and 14 days). The knockout of PTEN also promoted the regeneration of RGC axons. This indicates that AAV-mediated PTEN knockout effectively promotes RGC axonal regeneration. AAV-mediated downregulation of the PTEN gene has been successfully applied in clinical therapies for Leber congenital amaurosis caused by RPE65 gene defects. In RPE65-deficient dog models, subretinal injection of AAV2 vectors delivering the normal RPE65 gene restored photoreceptor function, improved behavioral visual responses, and showed no significant toxicity or retinal structural abnormalities during long-term follow-up (up to 6 years) (Bucher et al., 2021; Botto et al., 2022).

### Nanoparticle-based drug delivery systems

Nanoparticles can penetrate the retinal barrier and further release the drugs to the site of injury. Nanostructures such as nanowires or nanotubes can serve as scaffolding materials, mimicking the structure of natural neural sheaths, and thereby facilitating ONR (Manoukian et al., 2021). Recently, Zuidema et al. (2020) employed the principle of electron adsorption to precisely load the small molecule bpV into porous silicon nanoparticles (pSiNPs). Next, the pre-loaded bpV-pSiNPs are uniformly dispersed within a polylactic-co-glycolic acid (PLGA) nanofiber matrix using airbrushing, creating a hybrid sustained-release system. In this case, pSiNPs provide an alternative approach to implanting water-soluble drugs into the PLGA fiber structures. Kim et al. (2016) employed mesoporous silica nanoparticles (MSNs) functionalized with carboxylate groups for delivering bpV. The mesoporous structure in the MSNs enables stable adsorption of bpV molecules on the mesopore walls, while the chemical modification on the surface of the MSNs provides a sustained release mechanism. Further, MSNs exhibit a high efficiency of absorption by neurons, reaching 85%. At present, Yu et al. (2020) found that PTEN by bpv inhibition in the fetal mouse cortical neuronal hydraulic shock damage model can regulate autophagy and promote damaged neuronal repair. However, BPV currently remains confined to the mouse experimental stage, and both the efficacy and safety of using BPV to inhibit PTEN for the treatment of optic nerve disorders still require further validation through large mammalian studies or clinical trials.

### Coordinated strategies for optic nerve regeneration

A coordinated strategy to jointly modulate multiple axon growth-associated signaling pathways may elicit a stronger and more durable ONR. By concurrently deleting PTEN and SOCS3 (Sun et al., 2011; Bei et al., 2016; Mak et al., 2020), researchers can achieve upregulation of the mTOR and JAK/STAT3 pathways in RGC, synergistically promoting ONR. In the study by Mak et al. (2020), Cre recombinase and surgical optic nerve crush were employed in three experimental groups: PTEN knockout mice, SOCS3 knockout mice, and dual PTEN/SOCS3 knockout mice, while the control group just received optic nerve crush alone without genetic manipulation. After the dual knockout of PTEN and SOCS3, the axonal regeneration rate in the experimental group was significantly higher than that in the PTEN single-knockout mice. Additionally, the survival ability of RGCs in the dual-knockout mice was markedly enhanced. These findings demonstrate that simultaneous knockout of PTEN and SOCS3 significantly enhances both neuroprotection and axonal regeneration, highlighting its robust potential in promoting optic nerve repair (Mak et al., 2020). Previous studies have shown that Zn²⁺ can harm ONR. Therefore, by eliminating Zn²⁺ while deleting PTEN (Li et al., 2017), one may further augment the stimulation of the PI3K/AKT signaling route to a great extent. Ocular inflammation facilitates ONR through the release of oncomodulin, which elevates anti-apoptotic and growth protein transcription. By simultaneously deploying PTEN deletion, ocular inflammation induction, and cAMP bolstering (Kurimoto et al., 2010), one study applied a tripartite approach that further elicited RGC survival and regeneration to a heightened degree. However, the coordinated strategy remains confined to the mouse experimental stage, and both its safety and efficacy still lack further validation through large mammalian studies or clinical trials.

## Safety

Focal points of our review: Can we maintain adequate safety while unleashing the potential of this potent growth-promoting pathway? As we know, PTEN is a crucial tumor suppressor gene. Li et al. (2022a) expressed concerns that deleting PTEN may lead to numerous side effects. The loss of the PTEN gene in certain cellular populations has been shown to result in significant anatomical alterations (Kwon et al., 2001; Marino et al., 2002), including enlarged neuronal and tissue growth, macrocephaly, hydrocephalus, and tumor formation (Keng et al., 2012). These changes are often accompanied by behavioral abnormalities such as seizures, ataxia, and autism spectrum behaviors (Backman et al., 2001; Kwon et al., 2001; Fraser et al., 2004). Fortunately, even the enduring deletion of PTEN in mature neurons does not manifest detrimental consequences (Gutilla et al., 2016; Gallent and Steward, 2018). Gutilla et al. (2016) found no significant pathological changes, such as tumor formation and necrosis. Under hematoxylin-eosin staining, the overall tissue structure remains stable, with only a few alterations observed in cortical thickness and cell size. Furthermore, during the extensive 18-month follow-up period, no serious anatomical or behavioral abnormalities were observed, and normal exploratory behavior was observed in the open field (Gutilla et al., 2016; Gallent and Steward, 2018). Here, we have systematically reviewed the adverse effects induced by loss of PTEN across diverse cellular populations *in vivo* (**Additional Table 3**). We observed a common factor among these operations that leads to severe adverse reactions: the knockout of PTEN during embryonic development. Based on this observation, we hypothesize that selectively deleting the PTEN gene in mature neurons of adult individuals may help avoid these side effects.

**Additional Table 3:** Side effects induced by PTEN deletion in mouse embryos

Model	Approach	Side effect	Reference
C57BL/6 mouse embryos	Delete the PTEN locus within the murine cerebellum and dentate gyrus	(1) Macrocephaly, and gradually compresses the brainstem, inducing hydrocephalus. (2) The cerebellar granule cell and molecular layers demonstrated significant hyperplasia with aberrant positioning of cells. (3) Seizures increase in frequency and duration with time. (4) Mortality transpired between 9 and 48 wk of life.	Kwon et al., 2001
Mouse embryos	Delete the PTEN locus within astrocytes	(1) The brain exhibited progressive macrocephaly, particularly affecting the cerebellum. (2) Seizures manifested in 20% of mice at 10 wk of age, with 40% mortality before 50 d of life.	Backman et al., 2001; Fraser et al., 2004
Mouse embryos	Delete the PTEN locus within OPCs	(1) Macrocephaly, fibrosis in white matter regions, which later develop into necrosis and cyst formation. (2) Abnormally generate intermediate neurons.	Maire et al., 2014
C57BL/6 mouse embryos	Delete the PTEN locus within the hippocampus	(1) Developmental abnormalities: Disorganized hippocampal structure, losing the normal delicate laminar organization; cerebellum also showed migration defects. (2) Loss of cellular polarity: The axons of granule cells grew into the molecular layer, losing the normal unipolarity. (3) Behavioral abnormalities: Increased anxiety, reduced social interaction, and spontaneous epileptic seizures occurred.	Zhou et al., 2009
Mouse embryos	Delete the PTEN and NF1 loci within the hippocampal Schwann cells	(1) Reduced longevity: The median survival rate was only 15 d. (2) Tumor formation: within the spinal nerve roots, cranial nerves, and ganglia. (3) Developmental defects: Aberrant migration of hippocampal and cerebellar granule cells. (4) Augmented malignancy: High-grade peripheral nerve sheath tumors.	Keng et al., 2012

This table systematically summarizes severe adverse effects observed in murine embryonic models following PTEN deletion. Key findings include macrocephaly, hydrocephalus, and neuronal hyperplasia in cerebellar/dentate gyrus PTEN-knockout embryos; progressive macrocephaly and seizures in astrocyte-specific PTEN-deleted mice; white matter fibrosis and paraplegia in oligodendrocyte precursor cell PTEN-deficient models; disorganized hippocampal structure and epileptic seizures in hippocampal PTEN-knockout embryos; and peripheral nerve sheath tumors in combined PTEN/NF1-deleted Schwann cells. Notably, these severe phenotypes stand in sharp contrast to the safety profiles observed in adult neuronal PTEN deletion models, indicating developmental stage-specific risks. Directly targeting the embryonic nervous system may trigger irreversible damage, while modulating PTEN in mature neurons holds greater therapeutic potential. NF1: Neurofibromin 1; OPCs: oligodendrocyte precursor cells; PTEN: phosphatase and tensin homolog.

## Limitations

The review systematically reviews the potential mechanisms by which PTEN downregulation promotes all five key stages of ONR (stress response, growth navigation, nerve regeneration, synaptic reconstruction, and remyelination). Building on this mechanistic foundation, the article discusses the safety concerns and cutting-edge techniques related to PTEN downregulation in neurons. However, the review still has certain limitations. Most of the studies reviewed in this article are focused on small mammals such as mice. There are differences between human optic nerves and those in small mammals, making it difficult to specifically and accurately elucidate the mechanisms by which PTEN promotes ONR in humans. Consequently, the mechanistic role of PTEN downregulation in ONR necessitates further exploration in large mammalian models and clinical trials.

Additionally, the safety profile of PTEN downregulation requires thorough validation. As a tumor suppressor gene, PTEN downregulation may induce neoplastic transformation risks. Although studies cited in this review demonstrate that PTEN deletion in mature neurons shows no significant adverse effects, current evidence predominantly derives from small mammalian models. This limitation necessitates comprehensive validation across evolutionary scales in phylogenetically diverse organisms such as non-human primates to definitively establish its safety profile.

While this article elucidates the specific roles of PTEN in various stages of ONR, it does not comprehensively explain the effects of PTEN in different cell types. There are currently no articles indicating a direct link between PTEN and clinically significant optic neuropathies, including optic nerve atrophy and papilledema; any potential hypotheses can only be inferred from other disease models. Further research is still needed to confirm the possible connections between PTEN and these diseases. Additionally, our review has certain limitations. Some regenerative effects in **Additional Table 2** lack comparability due to differences in methodologies, such as variations in protocols, control selection, and specimen and reagent selection across studies. Therefore, we recommend standardizing procedures to facilitate the evaluation of results.

## Conclusions and Prospect

This review systematically discusses the structure-function analysis of the cellular functions of PTEN and its role in regulating downstream signaling pathways of ONR. We have summarized the novel techniques for deleting and inhibiting PTEN expression. The benefits, drawbacks, and outcomes of applying diverse methods are also reviewed. We have discussed the safety issues arising from PTEN deletion and provided evidence that deleting PTEN in mature neurons of adult individuals does not lead to severe adverse reactions.

This review mainly discusses the effects of the PTEN downregulation on ONR. During the stress response period, PTEN downregulation mainly promotes microglial polarization as well as proliferation, phagocytosis, and neural scar formation. During the growth navigation period, the downregulation of PTEN promotes axon regeneration by promoting microtubule polymerization and axon outgrowth. During the nerve regeneration period, PTEN downregulation can facilitate mitochondrial transport to the injured sites to meet energy requirements and calcium storage and limit reactive oxygen species production by promoting mitophagy in the damaged mitochondria. During the remyelination period, downregulation of PTEN promotes axonal extension and boosts synapse formation. Besides, PTEN downregulation can also promote microglial cell microtubular polymerization and stability to optimize its cytoskeletal structure, thus facilitating the phagocytosis of damaged synapses and making room for the establishment of new synapses. Finally, during the remyelination period, PTEN downregulation regulates microglia and OPCs, accelerating the remodeling of myelin at injured sites. However, PTEN knockout does not contribute to complete ONR, possibly due to limitations in myelin regeneration (Park et al., 2008). Based on this, we propose a hypothesis that the combination of PTEN and GPR17 inhibitors may achieve complete ONR. We hope that future experiments will combine the mechanisms promoting remyelination with the downregulation of PTEN to verify our hypothesis.

How to target PTEN remains challenging. Traditional bpV induces off-target effects upon other phosphatases, leading to serious side effects. This paper comparatively evaluated diverse emerging techniques for PTEN loss, discussing the merits and effects of PAPs, CRISPR-dCas9, sdRNA, AAV, nanoparticles, and coordinated strategies. The considerable potential was shown to advance the field toward high specificity, efficiency, rapid action, slow release, and cost-effectiveness. Further refinement of these methods may optimize safe and selective PTEN deletion for regenerative application. Some scholars have raised concerns that deleting PTEN may lead to numerous side effects, with reports of tumors, hydrocephalus, epilepsy, and anxiety. However, other studies have observed only modest neurogenesis and mild inflammation, without severe side effects. Through a cross-comparative appraisal of diverse models, methodologies, and outcomes, we posit that significant off-target effects arose from deletions occurring during the embryonic stage, impacting various tissue types. Therefore, we hypothesize that deleting PTEN solely within mature neurons of adult individuals would not induce significant pathological or behavioral abnormalities. Continued rigorous preclinical evaluation is essential to substantiate the safety case for targeted neuronal PTEN modulation as a regenerative strategy.

Focusing regenerative research on the optic nerve can lay the foundation for approaches applicable to the central nervous system. This review discusses the structure and downstream signaling pathways of PTEN and clarifies how PTEN loss promotes ONR. However, some areas remain underexplored, and we recommend that future research investigate 1) mechanisms to concurrently promote remyelination and ONR following PTEN loss, 2) the development of novel gene delivery vectors, 3) the application of combinatorial therapeutic approaches, and 4) a more systematic evaluation of safety concerns before clinical translation regarding targeted PTEN loss in the mature optic nerve.

## Additional files:

***Additional Table 1:*** *Functional regulation strategies and delivery systems for phosphatase and tensin homolog modulation: strengths and weaknesses.*

***Additional Table 2:*** *Regenerative effects of phosphatase and tensin homolog functional regulation strategies and delivery systems.*

***Additional Table 3:*** *Side effects induced by PTEN deletion in mouse embryos.*

## Data Availability

*All relevant data are within the paper and its Additional files*.
